# Editorial: *In-vitro*, *in-vivo*, and *ex-vivo* models of ischemia-reperfusion injury in lung transplantation

**DOI:** 10.3389/fimmu.2026.1831454

**Published:** 2026-05-15

**Authors:** Chetan Pasrija, Nirmal S. Sharma, Ilker Iskender

**Affiliations:** 1 Department of Surgery, Johns Hopkins University School of Medicine, Baltimore, MD, United States; 2 Department of Medicine, Baylor College of Medicine, Houston, TX, United States; 3 Department of Thoracic Surgery, University Hospital Zurich, Zurich, Switzerland

**Keywords:** EVLP, lung transplantation, lung transplant - ischemia-reperfusion injury, primary graft dysfunction, primary graft dysfunction risk modification

## Introduction

Primary graft dysfunction (PGD) remains the leading cause of early morbidity and mortality following lung transplantation (LTx) and represents the clinical manifestation of ischemia-reperfusion injury (IRI). Despite advances in surgical technique, organ preservation, and perioperative care, PGD occurs in approximately 10–30% of recipients and is strongly associated with chronic lung allograft dysfunction and long-term mortality. The pathophysiology of IRI is complex, involving metabolic derangements during ischemia followed by inflammatory activation, oxidative stress, endothelial injury, and innate immune activation upon reperfusion.

Given the complexity of these processes, the study of IRI has relied on a spectrum of experimental approaches ranging from reductionist *in-vitro* systems to whole-organ *in-vivo* transplantation models and translational ex-vivo organ platforms. The studies presented in this Research Topic highlight how these complementary models provide distinct yet interconnected insights into the mechanisms underlying PGD and potential therapeutic strategies.

## *In-vitro* models: defining molecular drivers of injury

*In-vitro* systems provide an essential starting point for understanding the molecular mechanisms that initiate IRI. By isolating specific cellular pathways in endothelial or epithelial cells, investigators can dissect how hypoxia, oxidative stress, and inflammatory signaling interact to drive tissue injury.

In this context, Chen et al. reviewed the role of the cGAS–STING signaling pathway in transplant-associated IRI. This pathway links cellular damage to innate immune activation and represents a key mechanism through which sterile injury can initiate inflammatory cascades in transplanted organs. They highlight emerging experimental evidence suggesting that pharmacologic inhibition of the cGAS–STING pathway may attenuate IRI-associated inflammation and programmed cell death.

In another study, Kollareth and Sharma argue that precision-cut lung slices, by preserving the multicellular architecture and intercellular crosstalk of the lung, could serve as a tractable platform to interrogate precisely these questions, including whether heat stress produces durable reprogramming of the endothelial-immune interface, whether mitochondrial preservation contributes to the observed protection, and whether the resistance of alveolar macrophages to CS observed in the porcine model translates to human tissue.

These findings underscore how *in-vitro* mechanistic studies can identify molecular pathways that may ultimately become therapeutic targets. However, while cellular models are invaluable for defining signaling pathways, they cannot fully replicate the physiologic and immunologic complexity of transplantation.

## *In-vivo* models: reproducing the physiologic complexity of PGD

To capture the integrated physiologic and immunologic responses that occur during LTx, investigators frequently rely on *in-vivo* models. These systems allow the study of IRI within the context of donor tissue injury, recipient immune responses, and microvascular dysfunction.

Goda et al. utilized a murine LTx model and identified a novel inflammatory pathway driven by the extracellular matrix–derived peptide proline-glycine-proline (PGP). Moreover, pharmacologic neutralization of PGP attenuated graft injury via reduction of neutrophil infiltration. This study illustrates how pathways initially identified through molecular studies can be validated in physiologically relevant transplantation models, providing a crucial step toward translational application.

Glorion et al. provide the first *in vivo* characterization of how intraoperative corticosteroids (CS) act on distinct monocyte/macrophage subsets during the critical perioperative window. While CS potently suppressed co-stimulatory molecule expression on circulating CD16^+^ monocytic cells, resident alveolar macrophages were entirely refractory to this effect, leaving their antigen-presenting capacity intact and raising the possibility that a dominant resident driver of allogeneic T cell priming remains unsuppressed by current intraoperative dosing regimens. The complex signaling landscape of IRI simultaneously engages TLR4, NOD-like, and purinergic receptor pathways, likely rendering gene responses variably refractory to CS, while differential chromatin accessibility between circulating monocytes and tissue-resident macrophages, shaped by distinct ontogeny and epigenetic programming, may explain the striking subset-specificity observed. Whether donor preconditioning or combination strategies targeting the NF-κB pathway more durably could overcome this resistance remains clinically urgent.

These findings highlight how *in-vivo* models enable investigators to identify complex inflammatory networks that would be difficult to appreciate in reductionist systems.

Jenkins et al. reviewed the current understanding of PGD and highlighted the growing role of modern preservation strategies, including ex vivo lung perfusion (EVLP) and other organ-conditioning approaches, in mitigating IRI. Furthermore, the authors emphasize that donor and recipient factors together shape the risk of PGD, highlighting the multifactorial nature of the condition.

## *Ex-vivo* models: bridging mechanistic insight and clinical application

While *in-vitro* and *in-vivo* systems provide critical mechanistic insights, ex-vivo organ platforms have emerged as an increasingly important tool for transplant research. Technologies such as EVLP allow donor lungs to be maintained under physiologic conditions outside the body, enabling detailed functional assessment and therapeutic intervention prior to transplantation.

Perfusate dialysis using hemofilter has been shown to improve EVLP homeostasis. De Wolf et al. has studied the impact of this strategy on gene expression profiles using a pig model and demonstrated increased accumulation of inflammatory cytokines in the perfusate and higher endothelial activation in lung tissue as well as increased pulmonary vascular resistance. The authors raised concerns on the future application of perfusate dialysis in clinical settings.

Parapanov et al. demonstrate that a brief hyperthermic pulse at 41.5 °C during EVLP comprehensively abrogates Src kinase activation, VE-cadherin phosphorylation, peroxinitrite formation, and endothelial biomarker shedding through converging mechanisms: direct HSP70-mediated Src inhibition, HSP27-dependent actin cytoskeleton stabilization, and inflammasome suppression blunting TNFα and IL-1β. Notably, the preservation of constitutive Hsc70 suggests that mitochondrial protection may represent an additional unexplored dimension, given HSP70’s known role in maintaining mitochondrial membrane integrity and preventing permeability transition pore opening. Whether part of the observed ROS attenuation reflects upstream mitochondrial preservation remains an important open question. While endothelial function post-transplantation was not directly evaluated, the attenuation of the PVR increase, which correlates with post-transplant PGD severity, is encouraging. These findings raise the possibility that thermal preconditioning during EVLP could represent a pharmacology-free strategy to actively condition the donor lung prior to implantation, rather than simply assessing it.

Nakata et al. summarized the recent advances in EVLP focusing on clinical utilization of the platform from assessment of questionable donor lungs to prolongation of preservation time and potential areas, such as gene therapy and regenerative medicine for future innovations.

In another overview, Ponholzer et al. focused on the methodological differences in EVLP protocols and strategies to optimize EVLP via advances in perfusate composition, perfusion and ventilation strategies, lung positioning, and perfusate treatment with antibiotics. They emphasized the importance of prolongation of EVLP times without compromising the quality of donor lungs as the next step.

Ex-vivo systems therefore serve as a critical bridge between mechanistic discovery and clinical translation. These platforms provide a unique opportunity to study lung injury in intact organs while maintaining experimental control over perfusion, ventilation, and therapeutic interventions.

## A translational framework for studying lung transplant IRI

Taken together, these studies highlight the complementary roles of *in-vitro*, *in-vivo*, and ex-vivo models in advancing our understanding of IRI in LTx.

*In-vitro* models allow investigators to identify and characterize molecular pathways that connect cellular injury to inflammatory activation. *In-vivo* transplantation models enable these pathways to be studied within the complex physiologic environment of the transplanted organ, revealing how inflammatory cascades and immune responses contribute to PGD. Finally, ex-vivo organ platforms provide a translational interface in which potential therapeutic interventions can be tested in intact organs prior to clinical application.

This integrated experimental framework mirrors the broader trajectory of translational transplant research: discovery of molecular mechanisms, validation in physiologic models, and eventual testing in organ-level systems that approximate the clinical environment.

## Future directions

Despite significant progress in understanding the mechanisms of ischemia-reperfusion injury, PGD remains a major challenge in LTx. Continued integration of molecular biology, immunology, and organ-level physiology will be essential for identifying therapeutic strategies capable of interrupting the cascade of injury triggered during organ procurement, preservation, and reperfusion.

Future studies should focus on translating mechanistic discoveries into targeted interventions that can be delivered during organ preservation or ex-vivo perfusion. Advances in organ preservation technology, combined with improved mechanistic understanding of IRI, offer the potential to significantly reduce the burden of PGD.

Ultimately, the studies presented here underscore the importance of leveraging complementary experimental systems to address the complex biology of IRI in LTx. By integrating insights from *in-vitro*, *in-vivo*, and ex-vivo models, the field moves closer to developing effective strategies to prevent PGD and improve long-term outcomes after LTx.

